# Proteomic Analysis of Differentially Expressed Plasma Exosome Proteins in Heat-Stressed *Holstein cows*

**DOI:** 10.3390/ani15152286

**Published:** 2025-08-05

**Authors:** Shuwen Xia, Yingying Jiang, Wenjie Li, Zhenjiang An, Yangyang Shen, Qiang Ding, Kunlin Chen

**Affiliations:** 1 Institute of Animal Science, Jiangsu Academy of Agricultural Sciences, Nanjing 210014, China; 2 Jiangsu Provincial Engineering Research Center of Precision Animal Breeding, Nanjing 210014, China; 3 College of Animal Science and Technology, Nanjing Agricultural University, Nanjing 210095, China

**Keywords:** exosomes, heat stress, differentially expressed proteins, *Holstein cows*

## Abstract

Heat stress in dairy cows, caused by high temperature and humidity during summer, has led to significant declines in milk production and severe economic losses for farms. Exosomes—extracellular vesicles carrying bioactive molecules—are critical for intercellular communication and immunity but remain understudied in heat-stressed *Holstein cows*. In this study, we extracted exosomes from three heat-stressed (HS) cows and three non-heat-stressed (Ctr) cows and employed proteomics to analyze plasma exosomes. We identified a total of 28 upregulated and 18 downregulated proteins in the HS group compared to the control group. Notably, we observed a significant upregulation of key protein groups, including cytoskeletal regulators, signaling mediators, and coagulation factors, alongside the downregulation of HP-25_1. These differentially expressed proteins demonstrate strong potential as heat stress biomarkers. GO and KEGG analyses linked the differentially expressed proteins to actin cytoskeleton regulation and endoplasmic reticulum pathways. Additionally, protein–protein interaction (PPI) analysis revealed the PI3K-Akt signaling pathway as a central node in the cellular response to heat stress. These findings establish plasma exosomes as valuable biospecimens, provide valuable insights into the molecular mechanisms of heat stress response, and may contribute to the development of precision breeding strategies for enhanced thermal resilience in dairy herds.

## Simple Summary

Heat stress in dairy cows during summer reduces milk production and causes economic losses. Using proteomics, we analyzed plasma exosomes from heat-stressed (HS) and non-heat-stressed (Ctr) cows. Among 46 differentially expressed proteins, cytoskeletal, signaling, and coagulation protein groups were significantly upregulated, and HP-25_1 was downregulated, suggesting their utility as heat stress biomarkers in dairy cattle. Bioinformatic analyses found these differentially expressed proteins enriched in actin cytoskeleton regulation, endoplasmic reticulum stress, and PI3K-Akt signaling pathways. In summary, this work highlights plasma exosomes’ utility for heat stress and provides valuable insights into the molecular mechanisms of heat stress response.

## 1. Introduction

Heat stress in dairy cows caused by high temperatures and humidity during summer has led to a significant negative impact on dairy cattle performance and serious economic losses for farms [1,2,3,4]. The molecular and cellular basis of heat stress on livestock has been widely investigated, including physiological and biochemical variations [5,6,7]. However, the mechanism and development process of an animal’s response to heat stress are still poorly understood. It is known that heat can pose stress to cells directly and thus reduce cell vitality and induce cell apoptosis [8]. However, the mechanisms of cell–cell interactions under heat stress are still poorly understood.

In recent years, exosomes, representing novel mechanisms of cellular communication, have attracted much attention [9]. Exosomes are nanosized extracellular vesicles that are secreted by living cells [10,11]. They have also been known to be regulators of cell-to-cell communication by exchanging their contents, including proteins, mRNA, miRNAs, and lncRNAs, between cells [12]. With this feature, many studies examined their role in response to a variety of disease pathophysiologies, especially those associated with immune responses and inflammation [13,14,15]. Currently, there is also much discussion about the physiological functions of exosomes in response to heat stress conditions [16,17]. Clayton et al. [18] reported that heat stress can drive certain heat shock proteins (HSPs), such as HSP27, HSP70, and HSP90, to accumulate within exosomes. Chen et al. [19] found that a variety of lncRNAs and miRNAs exist in exosomes released by vascular endothelial cells under heat stress, and they are mainly apoptosis-related. In cows, we have previously reported that the numbers of miRNAs were significantly differentially expressed in heat-stressed milk exosomes, and we further validated the idea that those milk-derived exosome miRNAs could increase mammary gland resistance to heat stress [20]. Therefore, understanding changes in heat-stressed cows’ exosome cargoes may provide insights into the mechanism of how animals cope with heat stress.

Exosomes have been discovered to have different origins with different compositions and functions, such as in blood, urine, milk, etc. [21,22]. Among these exosomes from different origins, plasma circulates exosomes from all types of cells and is thought to reflect how animals respond to environmental changes in a comprehensive way [21,22]. However, little is known about the plasma exosome content in heat-stressed *Holstein cows*.

Previous studies have demonstrated that acute heat stress induces significant changes in protein expression patterns. Yu et al. [23] reported an upregulated expression of HSP27, HSP70, and HSP90 accompanied by the activation of MAPK signaling pathways in pig jejunum following 3-day heat stress. Similarly, Pearce et al. [24] observed increased HSP70 mRNA and protein abundance under acute HS conditions. Building on these findings, the aim of this study is to investigate plasma exosome proteins in heat-stressed *Holstein cows* and their potential biological functions. Recent rapid developments in high-throughput and sensitivity screening in proteomics technology can provide an opportunity to comprehensively study the composition and function of exosome proteins. In the current study, we performed a comprehensive comparative proteomic profiling of plasma-derived exosomes from HS and Ctr cows. Moreover, we investigated the KEGG pathway and interaction of those differentially expressed proteins to further understand the mechanism of how animals cope with heat stress. This study can provide valuable insights into the molecular mechanisms of heat stress adaptation in dairy cattle and lay the foundation for improving heat stress resistance by selective breeding.

## 2. Materials and Methods

### 2.1. Sample Collection

Six healthy *Holstein dairy cows* with the same parity and lactation period were selected from the same dairy farm. Three of them were assigned to the heat stress group (HS), and samples were collected in August (summer); the other three were in the non-heat stress group (Ctr), and samples were collected in December (winter). During 3 consecutive days before sampling, the temperature–humidity index (THI), rectal temperature, and respiratory rate were recorded, as shown in Table 1. Blood samples (10 mL) were collected from each cow using the tail vein blood collection method and were placed in EDTA anticoagulant tubes. After mixing, samples were transported to the laboratory at a low temperature within 1 h. In the laboratory, the blood samples were centrifuged at 3000× *g* for 15 min at 4 °C to separate the plasma, which was stored at−80 °C for subsequent sequencing.

### 2.2. Plasma Exosome Isolation

The frozen plasma samples were thawed in a 37 °C water bath. Once dissolved, each sample was transferred to a new centrifuge tube and centrifuged at 10,000× *g* for 30 min at 4 °C. The supernatant was then transferred to another centrifuge tube and centrifuged at 10,000× *g* for 45 min at 4 °C. Large particles were removed by filtering the supernatant through a 0.45 μm filter. The filtered supernatant was collected into a new tube and ultracentrifuged at 100,000× *g* for 70 min at 4 °C. After discarding the supernatant, the pellet was resuspended in 10 mL of precooled 1×PBS and centrifuged again at 100,000× *g* for 70 min at 4 °C. The supernatant was removed, and the pellet was resuspended in 100 μL of precooled 1× PBS. Of this suspension, 10 μL was used for electron microscopy detection, 10 μL for particle size analysis, and 30 μL for protein extraction. The remaining exosomes were stored at −80 °C for subsequent experiments.

### 2.3. Identification of Plasma Exosomes

#### 2.3.1. Transmission Electron Microscopy

Ten microliters of exosomes were dropped onto a copper grid for precipitation for 1 min, and the excess liquid was blotted with filter paper. Then, 10 μL of uranyl acetate was added to the copper grid for staining for 1 min, and the surplus liquid was absorbed by filter paper, followed by air-drying at room temperature for 5 min. The morphology of exosomes was observed under a high-resolution electron microscope (HR-TEM, HT-7700, Hitachi, Chiyoda, Japan).

#### 2.3.2. Nanoparticle Tracking Analysis (NTA)

Ten microliters of exosomes were diluted three times, and the gradient-diluted samples were loaded for analysis. After sample detection using Zetasizer Nano S90 (Malvern, Worcestershire, UK), the particle size and concentration of exosomes were obtained.

#### 2.3.3. Western Blotting (WB)

Plasma exosomes were lysed using RIPA buffer at a low temperature for 30 min, and protein concentration was quantified with BCA kits. The exosome proteins were denatured in a metal bath at 100 °C for 10 min. A 30 ng protein sample was subjected to 12% SDS-PAGE electrophoresis, and the separated proteins were transferred onto a PVDF membrane. The membrane was blocked with 5% skim milk for 1 h and then incubated with primary antibodies (CD81, TSG101, CD63, an Calnexin; Proteintech, Rosemont, IL, USA) overnight at 4 °C. After washing the membrane three times with 1× TBST at room temperature for 10 min each, it was incubated with a secondary antibody (Proteintech) solution for 90 min at room temperature. Following three additional 10 min washes with 1× TBST, the membrane was treated with an ECL chemiluminescence solution and scanned in the infrared imaging system.

#### 2.3.4. Sample Processing

SDT buffer (4% SDS, 100 mM Tris-HCl, 1 mM DTT, pH 7.6) was used for sample lysis and protein extraction. Protein concentration was quantified using the BCA Protein Assay Kit (Bio-Rad, Hercules, CA, USA). Protein digestion with trypsin was performed using the filter-aided sample preparation (FASP) protocol. Briefly, 200 μg of protein was denatured in SDT buffer (4% SDS, 100 mM DTT, 150 mM Tris-HCl, pH 8.0), purified through 10 kDa filters using UA buffer (8 M urea, 150 mM Tris-HCl, pH 8.0), alkylated with 100 mM IAA (30 min, dark), and digested overnight with 4 μg of trypsin in 25 mM NH_4_HCO_3_ at 37 °C. Peptides were desalted (C18 cartridges), vacuum-concentrated, resuspended in 0.1% formic acid, and quantified by UV280 (an extinction coefficient of 1.1 based on Trp/Tyr content).

#### 2.3.5. Liquid Chromatography–Mass Spectrometry (LC–MS)

Samples were analyzed using an Easy-nLC HPLC system coupled to a Q-EXactive mass spectrometer (Thermo Fisher Scientific, Waltham, MA, USA), operating at a 300 nL/min flow rate with 0.1% formic acid in water (Buffer A) and 0.1% formic acid in 84% acetonitrile (Buffer B) as mobile phases. After equilibrating the system with 95% Buffer A, samples were loaded onto a PepMap100 C18 trapping column (Thermo Fisher Scientific, Waltham, MA, USA, 100 μm × 2 cm) and separated on an EASY C18-A2 analytical column (Thermo Fisher Scientific, Waltham, MA, USA, 75 μm × 10 cm, 3 μm). Mass spectrometry analysis was performed in positive ion mode with a 300–1800 *m*/*z* scan range, a 70,000 MS1 resolution at 200 *m*/*z*, a 1 × 10^6^ Automatic Gain Control (AGC) target, and a 50 ms maximum injection time, employing a 60 s dynamic exclusion. For each full scan, twenty MS2 spectra were acquired using HCD fragmentation with 2 *m*/*z* isolation windows, a resolution of 17,500, a collision energy of 30 eV, and a 0.1% underfill ratio.

#### 2.3.6. Label-Free Analysis of MaxQuant

The mass spectrometry raw data were processed using MaxQuant (v1.5.3.17) for label-free quantitative (LFQ) analysis, which applies peptide- and protein-level pairwise correction algorithms for multi-group comparisons. Relevant parameters are detailed in Table 2.

### 2.4. Bioinformatic Analysis

#### 2.4.1. Cluster Analysis of Phosphorylated Peptides

Hierarchical clustering analysis was performed using Cluster 3.0 (http://bonsai.hgc.jp/~mdehoon/software/cluster/software.htm, accessed on 28 July 2025) and Java Treeview (http://jtreeview.sourceforge.net, accessed on 28 July 2025). Euclidean distance was used for similarity measurement, and average linkage clustering (based on centroids) was selected for hierarchical clustering, with results visualized through heatmaps and corresponding dendrograms.

#### 2.4.2. Subcellular Localization

Protein subcellular localization was predicted using the multi-class SVM classification system CELLO (http://cello.life.nctu.edu.tw/, accessed on 28 July 2025).

#### 2.4.3. Annotation and Enrichment Analysis

Protein domain signatures were identified by searching sequences against the Pfam database using InterProScan software (v5.26–65.0). Gene Ontology (GO) annotation was performed with Blast2GO 6.0. Proteins were then BLAST-searched against the Kyoto Encyclopedia of Genes and Genomes (KEGG) database (http://www.genome.jp/kegg/, accessed on 28 July 2025) to obtain orthology IDs. Fisher’s exact test was used to identify enriched GO terms and KEGG pathways. Significantly enriched terms were identified using an adjusted *p*-value threshold of <0.05.

#### 2.4.4. PPI Analysis

PPI data were obtained from IntAct and STRING databases using gene symbols, and the resulting networks were analyzed using Cytoscape software (version 3.2.1).

### 2.5. Data Analysis

Data are presented as mean values ± standard deviation (SD). Statistical analysis was performed using Student’s *t*-test with GraphPad Prism 8.0.1 (La Jolla, CA, USA). A *p*-value < 0.05 was considered statistically significant.

## 3. Results

### 3.1. Identification of Pasma Exosomes

**Figure 1:**
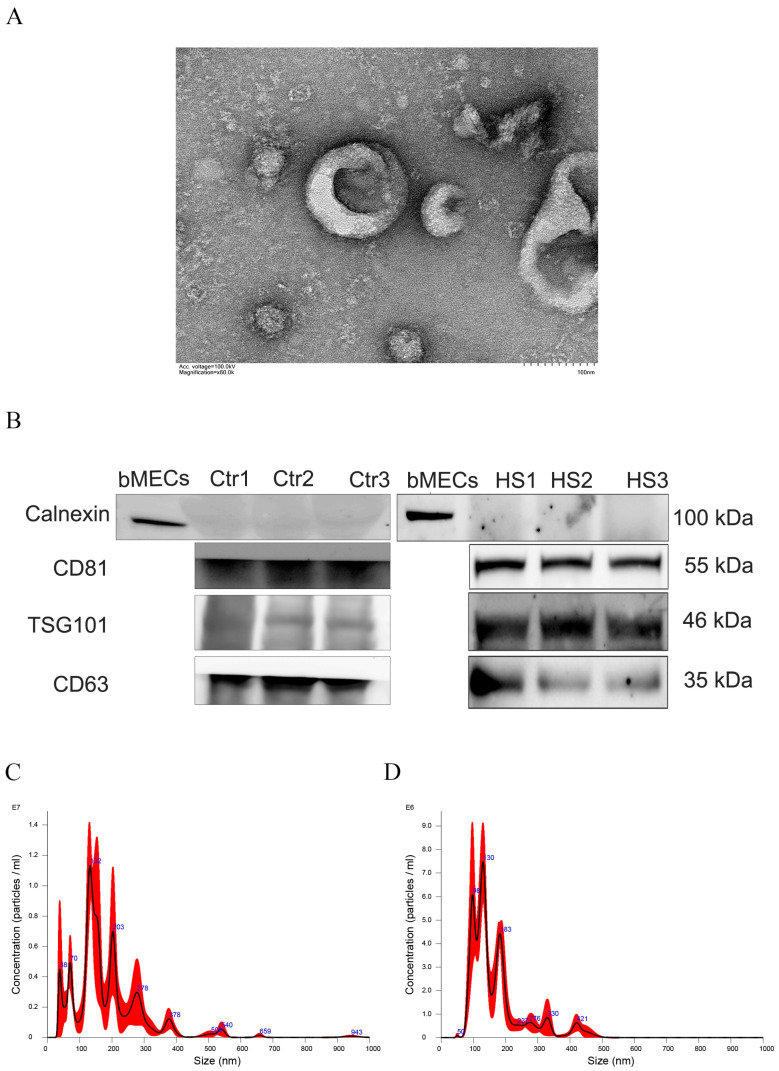
Isolation and characterization of plasma exosomes. (**A**) Transmission electron microscopy (TEM) image of plasma exosomes. (**B**) Western blot analysis of exosome protein markers (CD63, CD81, TSG101, and Calnexin C). BMECs denote bovine mammary epithelial cells. (**C**,**D**) Nanoparticle tracking analysis (NTA) of plasma exosomes.

Transmission electron microscopy analysis confirmed the successful isolation of exosomes, revealing characteristic cup-shaped vesicles with intact lipid bilayer membranes (Figure 1A). Nanoparticle tracking analysis demonstrated that most of the vesicles fell within the canonical exosome size range (30–200 nm), with significantly higher concentrations in the HS group (1.10 × 10^11^ ± 4.98 × 10^7^ participants/mL) compared to the Ctr group (6.27 × 10^10^ ± 3.0 × 10^7^ participants/mL; Figure 1C,D). In addition, compared with bovine mammary epithelial cells (BMECs), marker proteins CD81, CD63, and TSG101 were detected in exosomes, while the cell membrane protein Calnexin was not detected (Figure 1B), indicating that the extracted exosomes are free from somatic cell contamination. The above results suggest that high-purity exosomes were extracted and can be used for subsequent analysis in this study.

### 3.2. Identification of Plasma Exosome Proteins and Peptides

**Figure 2:**
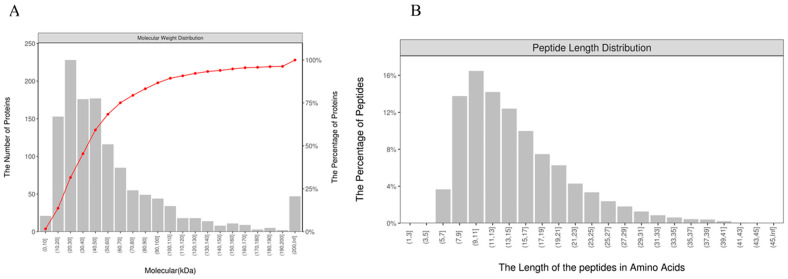
Distribution of protein size and peptide length. (**A**) Protein size distribution in plasma exosomes. (**B**) Peptide length distribution in plasma exosomes.

Mass spectrometry identified proteins that were predominantly in the range of 10–110 kDa (Figure 2A). To further verify the appropriateness of the selected protease, the length of the peptide was identified. The peptide segment lengths were mostly between 7 and 23 amino acids, which is in line with the reasonable range of peptide length (Figure 2B). This also indicates that the enzyme digestion is sufficient and that the data is reliable.

### 3.3. Analysis of Differential Proteins in Plasma Exosomes

**Figure 3:**
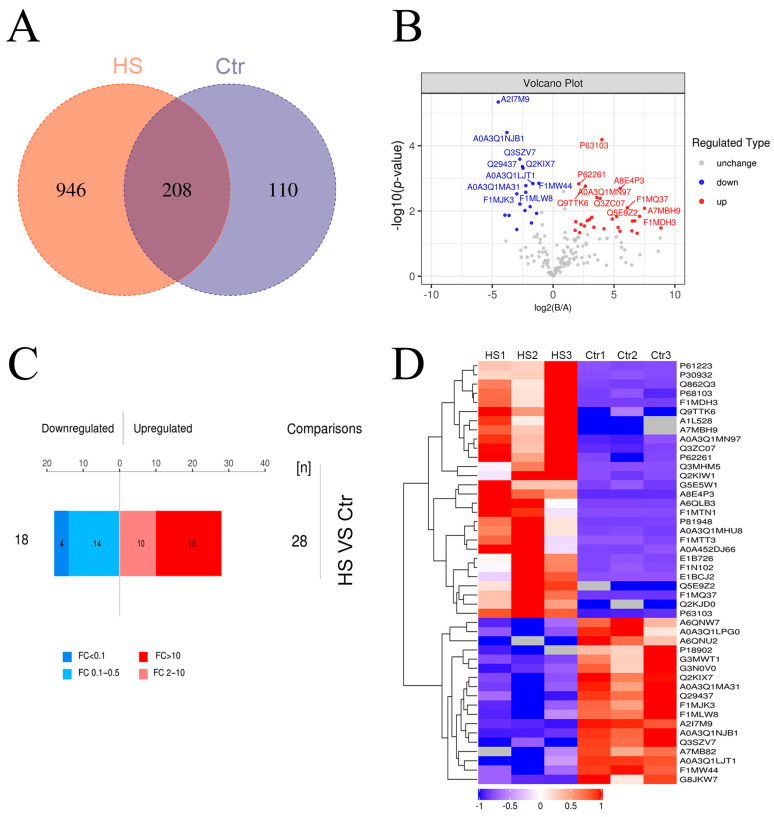
Heatmap and volcanic map analysis of proteins differentially expressed by HS and Ctr groups. (**A**) Venn diagram of different proteins in the Ctr group and the HS group. (**B**) Volcano plot of differentially expressed proteins in the HS and Ctr groups. The downregulated proteins (FC < 0.5 and *p* < 0.05) are highlighted in blue, upregulated proteins are highlighted in red (FC > 2.0 and *p* < 0.05), and gray represents the non-significant proteins (FDR > 0.05). (**C**) Upregulated and downregulated protein quantity between HS and Ctr groups. (**D**) Heatmap of differentially expressed proteins in the HS and Ctr groups.

A total of 1264 proteins were identified. Specifically, 1154 proteins were detected in the HS group, 318 were detected in the Ctr group, and 208 were shared by both groups (Figure 3A). In this study, proteins with fold changes (FCs) greater than 2 or less than 0.5 were considered to be differentially expressed proteins between HS and Ctr. When the *p*-value was <0.05, a total of 46 proteins were significantly differentially expressed between HS and Ctr (Table 3; Figure 3B). Out of these 46 proteins, 28 proteins were significantly upregulated, and 18 proteins were significantly downregulated. Furthermore, we raised the FC threshold and found that 18 proteins were upregulated (FC > 10 and *p*-value < 0.05) and four proteins were downregulated (FC < 1 and *p*-value < 0.05).

To analyze the expression patterns of samples between HS and Ctr, differentially expressed proteins were subjected to hierarchical cluster analysis. As shown in Figure 3D, the significantly differentially expressed proteins enabled effective discrimination between HS and Ctr.

### 3.4. Subcellular Localization Analysis

**Figure 4:**
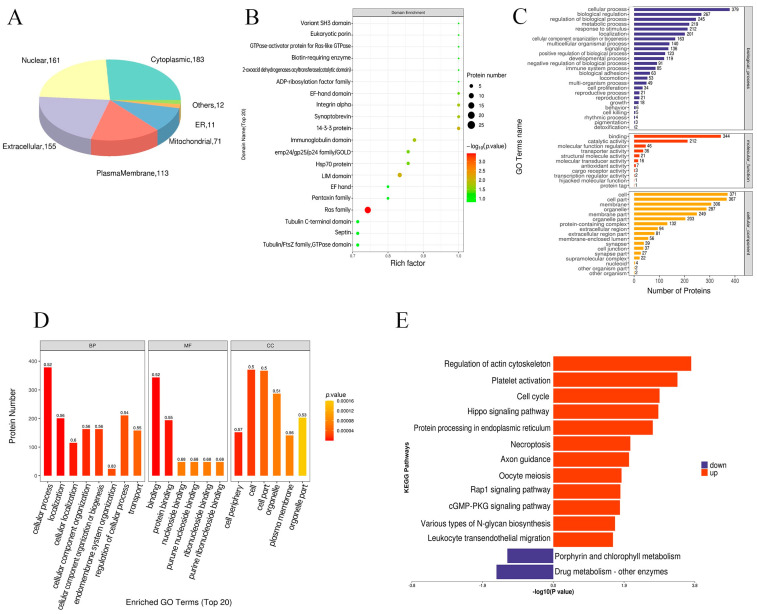
Bioinformatics analysis of differentially expressed proteins in plasma exosomes derived from HS and Ctr groups. (**A**) The distribution of differentially expressed proteins in sub-organelles. (**B**) The distribution of differentially expressed protein structural domains. (**C**) GO enrichment of differentially expressed proteins. (**D**) Top 20 most enriched terms in GO enrichment. (**E**) KEGG pathway for differentially expressed proteins.

The differentially expressed proteins were predominantly localized to cytoplasmic (25.92%), nuclear (22.80%), extracellular (21.55%), and plasma membrane (16.01%) compartments, with additional minor distributions in other organelles (Figure 4A). Domain enrichment identified Ras family proteins, including immunoglobulin domains V-set and C1-set, as predominant features (Figure 4B).

### 3.5. GO and KEGG Enrichment Analyses for Differentially Expressed Proteins

The functional categorization of differentially expressed proteins was performed using Blast2Go. The results showed that these proteins were primarily involved in biological processes such as cellular processes, localization, cellular localization, cellular component organization, and cellular component organization or biogenesis. In terms of molecular functions, they were enriched in binding activities, including protein binding, ribonucleoside binding, GTP binding, and nucleoside binding. For cellular components, the proteins were mainly distributed in the cell peripheries, cells, cell parts, organelles, and plasma membranes (Figure 4C,D).

KEGG pathway analysis revealed that upregulated proteins in the HS group were significantly enriched in pathways related to actin cytoskeleton regulation, Hippo signaling, protein processing in the endoplasmic reticulum, Rap1 signaling, and MAPK signaling (Figure 4E), suggesting key roles in the specific physiological state of the HS group. Downregulated proteins were mainly enriched in drug metabolism and porphyrin/chlorophyll metabolism pathways (Figure 4E), potentially related to altered metabolic homeostasis.

### 3.6. PPI Network of Differentially Expressed Proteins

**Figure 5:**
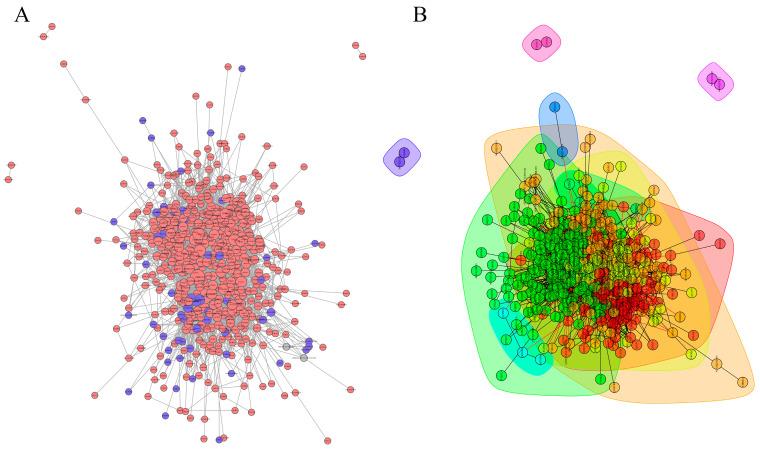
Analysis of HS group and Ctr group protein–protein interaction map. (**A**) Network diagram of interaction in plasma exosome proteins. The circular nodes represent differentially expressed proteins, and lines denote protein–protein interactions. The color of the circles indicates the expression difference in proteins, where blue denotes downregulation and red denotes upregulation in HS compared to Ctr. (**B**) Functional classification diagram of plasma exosome protein interaction network. Proteins with high aggregation degrees in the interaction network were partitioned into distinct clusters. Each cluster is represented by a unique color.

**Figure 6:**
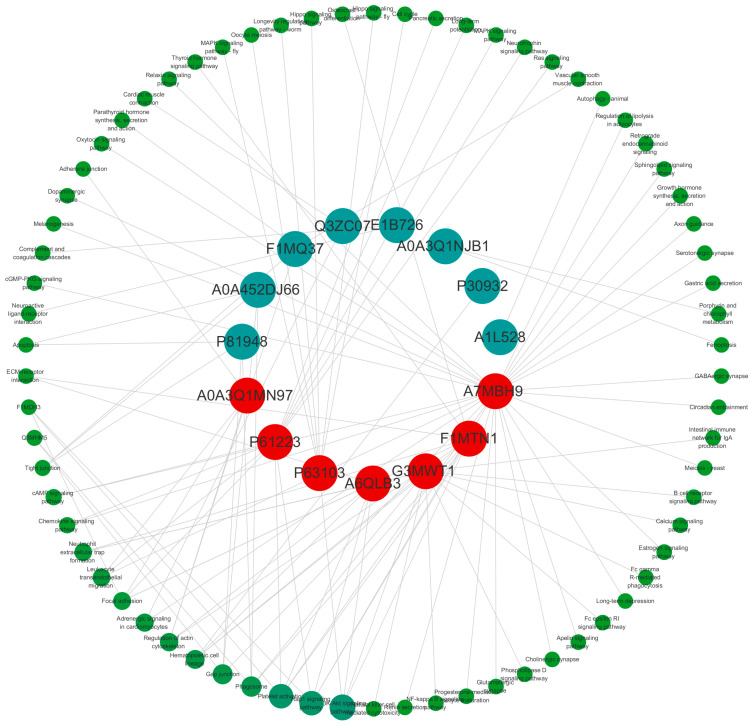
Key differentially expressed proteins and their associated signaling pathways. The color of the inner circles indicates the degree of enriched pathways, where red denotes the most enriched and blue denotes the least enriched. The outer green circle indicates protein-associated signaling pathways.

The PPI network of differentially expressed proteins was constructed based on the STRING and IntAct databases and revealed that five clusters of differentially expressed proteins exhibited potential direct or indirect interactions (Figure 5A,B). Interaction analysis of proteins with FC > 10 or <1 showed that A0A3Q1MN97, P61223, P63103, and others were most enriched in signaling pathways. Among these, the PI3K-Akt signaling pathway, Rap1 signaling pathway, and platelet activation pathway exhibited the highest betweenness centrality (Figure 6), indicating that they are core regulatory networks for differentially expressed proteins. The findings indicated enhanced biological connections among these proteins, though their biological functions require further validation.

## 4. Discussion

Heat stress represents a major challenge in modern dairy production systems, significantly impacting animal welfare and productivity [25,26]. A comprehensive understanding of the mechanisms underlying heat stress in dairy cows will facilitate future improvements for advancing breeding strategies and management practices. Growing evidence indicates that exosomes play a pivotal role in mediating cellular responses to heat stress [20]. Plasma exosomes mediate intercellular communication by transporting proteins, nucleic acids, and lipids, and their cargo proteins play critical roles in regulating physiological and pathological processes, including stress responses [21,22]. However, there remains a gap regarding the systematic characterization of plasma exosome proteomics and its functional implications for heat stress resistance in dairy cattle.

With the employment of proteomic approaches in the current study, we have identified the differentially expressed proteins resulting from the HS and Ctr groups. Specifically, we identified 46 differentially expressed proteins, with 28 showing significant upregulation and 18 demonstrating downregulation in the HS group (Table 3). Intriguingly, many of these proteins have established associations with heat-related responses, confirming the biological relevance of our findings.

Among the upregulated proteins, several families emerged as potentially pivotal in the cellular response to heat stress, including cytoskeletal proteins (i.e., tubulin isoforms, including TUBA1D, TUBA4A, TUBB4B, and TUBB5), signaling proteins (YWHAZ and YWHAE 14-3-3 proteins, as well as ITGB3 and ITGA2B integrins), and coagulation proteins (i.e., F8 and F12). Tubulin, the main component of microtubules, is essential for maintaining the cytoskeleton’s structure. Previous studies have shown that under heat stress, the upregulation of α-tubulin expression enhances microtubule stability, allowing cells to preserve their structural integrity and functionality in the face of thermal challenges [27]. Additionally, oxidative stress preconditioning increases intracellular levels of both HSP90 and tubulin, suggesting a cooperative protective role of tubulin with HSP90 during oxidative stress adaptation [28]. In the context of heat stress, the upregulation of tubulin isoforms in our study may disrupt the normal cytoskeletal dynamics, potentially impairing cellular functions such as intracellular transport and cell division.

The 14-3-3 proteins are highly conserved intracellular adaptors involved in multiple biological processes, including signal transduction, cell cycle regulation, and apoptosis [29]. In *Drosophila* cells, 14-3-3ζ is upregulated under heat stress via heat shock transcription factor-mediated mechanisms, where it acts as a molecular chaperone to dissolve heat-aggregated proteins [30]. The upregulation of 14-3-3 proteins in our HS group supports their conserved role in coordinating cellular responses to thermal stress, likely by stabilizing stress-related signaling cascades or preventing protein misfolding. Integrins, as cell-surface receptors, play crucial roles in cell–extracellular matrix interactions, cell adhesion, and signaling. Integrin-linked kinases have been shown to modulate thermotolerance in *Caenorhabditis elegans* through neuronal control of HSF-1 [31]. The upregulation of ITGB3 and ITGA2B in the HS group may disrupt these processes, affecting cell–cell and cell–matrix communication, which are essential for maintaining tissue homeostasis during heat stress.

Coagulation factors, which are directly involved in blood coagulation, are also affected by heat stress [32,33]. Heat stress can impact coagulation factors through various pathways, such as damaging vascular endothelial cells, activating mononuclear macrophages, and inducing the release of inflammatory factors [34]. The upregulation of F8 and F12 in our study may contribute to the altered coagulation function often observed in heat-stressed animals, potentially increasing the risk of bleeding or thrombosis.

Eukaryotic elongation factor-1 α1 (EEF1A1), another upregulated protein, emerged as a critical orchestrator of the heat stress response. EEF1A1 is known to regulate protein synthesis and immune function [35]. Recent studies have established its role as a central coordinator of the heat shock response [36]. Under thermal stress conditions, *EEF1A1* coordinates cellular adaptation by recruiting transcription factors to activate the transcription of heat shock genes and stabilizing heat shock protein (HSP) mRNAs, such as *HSP27*, *HSP60*, and *HSP70* [36]. These HSPs are essential for protein refolding, oligomeric assembly, and immune modulation under stress conditions [37,38,39]. Therefore, *EEF1A1* may facilitate nuclear export of HSP mRNAs and their delivery to ribosomes, enabling rapid cellular adaptation to proteotoxic stress. This dual role positions *EEF1A1* as a key orchestrator of the heat shock response, ensuring proteome stability during heat stress.

In the downregulated protein set, HP-25_1 (HP-25 homolog 1) stood out as a Bos taurus-specific protein. Our observation of HP-25_1 downregulation in the HS group aligns with previous 2-DE studies on dairy cow plasma, which linked adiponectin upregulation to heat stress acclimation—a potential feedback mechanism for thermal adaptation [40]. HP-25_1′s species specificity to cattle and its inverse regulation with adiponectin in our study suggest a role in mediating heat stress responses in ruminants. Further investigation into the functional relationship between HP-25_1 and adiponectin could uncover novel pathways underlying heat acclimation in dairy cows.

Notably, KEGG pathway enrichment analysis of the differentially expressed proteins revealed that the actin cytoskeleton pathway was primarily enriched (Figure 4E). These findings align with previous research where heat-induced downregulation of tight junction proteins and actin regulators impairs intestinal barrier function [41]. Additionally, studies in human cancer cells have identified a novel interaction between HSP27 and the actin cytoskeleton, which collectively regulate cellular motility [42]. Together, these results suggest a conserved cytoprotective mechanism in which heat-stressed cells dynamically reorganize their actin cytoskeleton to counteract thermal damage, potentially by stabilizing cellular structures or modulating stress-responsive signaling pathways.

In addition to the actin cytoskeleton, the endoplasmic reticulum (ER) pathway was also significantly enriched and may be associated with heat stress (Figure 4E). As the primary site for protein synthesis, folding, and calcium storage, the ER is highly sensitive to thermal stress [43,44,45]. Heat-induced protein misfolding and unfolded protein accumulation disrupt ER homeostasis, while potentially altering calcium balance, collectively triggering ER stress [46,47]. Thus, proper ER protein processing is essential for cell survival during heat stress, as impaired function reduces thermotolerance and compromises animal health and productivity. In broilers, chronic heat stress decreases GRP78 expression in the gastrocnemius muscle [48], suggesting that ER dysfunction may underlie heat-induced muscle impairment and growth delays. While this study establishes a link between exosome proteins and ER pathway activation, further research is needed to clarify how these proteins specifically modulate ER stress responses in HS-exposed cells.

PPI analysis revealed the PI3K-Akt signaling pathway as the highest-scoring node in betweenness centrality (Figure 6). Interestingly, emerging evidence demonstrates that heat stress potently activates the PI3K-Akt signaling pathway across multiple biological systems, with significant implications for cellular homeostasis. A recent study in dairy goats revealed that thermal stress upregulates *p*-Akt and *p*-PI3K levels while increasing the Bax/Bcl-2 ratio, ultimately triggering apoptosis in spermatocytes and early spermatids [49]. Additionally, another study has indicated that the PI3K/mTORC2-dependent AKT pathway has been identified as a critical survival mechanism for hepatocellular carcinoma cells during thermal ablation-induced heat stress [50]. Our findings corroborate this pathway′s central role in heat stress responses, suggesting that its activation represents an evolutionarily conserved mechanism. However, additional studies are required to elucidate the precise triggers of pathway activation under heat stress conditions.

In summary, this study provides novel insights into dairy cow heat stress responses through plasma exosome proteomics, identifying a suite of differentially expressed proteins in response to heat stress and key stress-response pathways. These results not only enhance our understanding of heat stress resistance but also have direct translational potential, guiding future breeding programs to select for heat-tolerant genotypes and informing management practices to mitigate heat stress impacts on animal welfare and productivity. However, two key limitations must be addressed. Firstly, while the identified proteins and pathways suggest functional roles, the study relies on correlative data from proteomic analysis. Mechanistic validation, such as in vitro functional assays or gene knockdown experiments, is needed to confirm their causal involvement in heat stress adaptation. Additionally, the current research does not explore the temporal dynamics of exosome protein expression during heat stress exposure, which could reveal critical time points for intervention. Future studies should be conducted to bridge these gaps to facilitate practical applications in dairy production systems.

## 5. Conclusions

In this study, we identified heat-responsive proteins in dairy cattle, with a distinct upregulation of cytoskeletal, signaling, and coagulation-related proteins alongside the downregulation of HP-25_1. Three key pathways (actin cytoskeleton regulation, ER stress, and PI3K-Akt signaling) were significantly altered. These findings provide valuable insights into the molecular mechanisms underlying heat stress adaptation in dairy cattle, while highlighting potential biomarkers for early heat stress detection and improving heat tolerance through selective breeding programs. However, the precise mechanisms through which these proteins contribute to heat stress require further investigation.

## Figures and Tables

**Table 1:** Parity, lactation days, record of temperature–humidity index (THI), rectal temperature, and respiratory rate with their standard deviation (SD) in HS and Ctr.

Group	Parity	Lactation Days (d)	Average Rectal Temperature/°C	Temperature and Humidity Index (THI)	Average Breathing Rate (Times/Min)
Ctr1	2	126	38.40 ± 0.22	55.41 ± 1.49	47 ± 2.03
Ctr2	2	128	38.43 ± 0.09	55.41 ± 1.49	52 ± 1.78
Ctr3	2	132	38.60 ± 0.08	57.87 ± 3.33	53 ± 2.10
HS1	2	125	39.40 ± 0.36	79.57 ± 0.89	106 ± 2.65
HS2	2	130	39.50 ± 0.24	77.44 ± 3.43	96 ± 2.00
HS3	2	131	39.83 ± 0.34	79.95 ± 3.32	108 ± 2.65

**Table 2:** MaxQuant identification and quantitative parameters.

Item	Value
Enzyme	Trypsin
Max missed cleavages	2
Main search	6 ppm
First search	20 ppm
MS/MS tolerance	20 ppm
Fixed modifications	Carbamidomethyl (C)
Variable modifications	Oxidation (M)
Database	uniport_Bovine_46480_20200217.fasta
Database pattern	Reverse
Include contaminants	True
Protein FDR	≤0.01
Peptide FDR	≤0.01
Peptides used for protein quantification	Used razor and unique peptides
Time window (match between runs)	2 min
Protein quantification	LFQ
Min. ratio count	1

**Table 3:** Differentially expressed proteins in HS and Ctr groups.

Upregulated					Downregulated				
Protein Symbol	Protein Name	Gene Name	FC *	*p*-Value	Protein Symbol	Protein Name	Gene Name	FC *	*p*-Value
F1MTN1	Integrin beta	ITGB3	465.04	0.0331196	A2I7M9	Serpin A3-2	SERPINA3-2	0.04	4.56 × 10^−6^
A7MBH9	G protein subunit alpha i2	GNAI2	181.86	0.0082595	G8JKW7	Serpin A3-7	SERPINA3-7	0.06	0.013396
F1MDH3	Talin 1	TLN1	137.56	0.0145823	A0A3Q1NJB1	Ceruloplasmin	CP	0.07	3.93 × 10^−5^
E1BCJ2	Complement factor H related 5	CFHR5	119.67	0.047979	G3MWT1	Ig-like domain-containing protein	IGLDCPs	0.08	0.013672
A1L528	small monomeric GTPase	RAB1A	104.68	0.0200544	A6QNW7	CD5 molecule-like	CD5L	0.1	0.006112
A6QLB3	Integrin subunit alpha 2b	ITGA2B	91.14	0.020363	F1MJK3	Uncharacterized protein	LOC506828	0.12	0.002985
Q3MHM5	Tubulin beta-4B chain	TUBB4B	88.96	0.0407212	P18902	Retinol-binding protein 4	RBP4	0.13	0.03687
F1MQ37	Myosin-9	MYH9	68.31	0.0079969	Q3SZV7	Hemopexin	HPX	0.15	0.000258
A8E4P3	Stomatin	STOM	45.32	0.0020229	Q29437	Primary amine oxidase, liver isozyme	LOC100138645	0.17	0.000435
P30932	CD9 antigen	CD9	45.12	0.0418379	Q2KIX7	Protein HP-25 homolog 1	LOC511240	0.18	0.000489
A0A452DJ66	Tubulin alpha chain	TUBA1D	40.44	0.0317772	G3N0V0	Ig-like domain-containing protein	IGLDCPs	0.2	0.009688
Q5E9Z2	Hyaluronan-binding protein 2	HABP2	37.37	0.0148506	A0A3Q1MA31	Inter-alpha-trypsin inhibitor heavy chain H4	ITIH4	0.21	0.001666
P81948	Tubulin alpha-4A chain	TUBA4A	29.09	0.017609	F1MLW8	Uncharacterized protein	-	0.21	0.002664
P61223	Ras-related protein Rap-1b	RAP1B	18.11	0.0350055	A6QNU2	IVL protein	IVL	0.27	0.007375
P63103	14-3-3 protein zeta/delta	YWHAZ	16.06	6.474 × 10^−5^	A7MB82	Complement C1q tumor necrosis factor-related protein 3	C1QTNF3	0.29	0.023276
Q3ZC07	Actin, alpha cardiac muscle 1	ACTC1	14.54	0.0041034	A0A3Q1LJT1	Ig-like domain-containing protein	IGLDCPs	0.32	0.001441
A0A3Q1MN97	Vinculin	VCL	12.05	0.0038576	A0A3Q1LPG0	Ig-like domain-containing protein	-	0.39	0.0118
E1B726	Plasminogen	PLG	10.19	0.0315644	F1MW44	Coagulation factor XIII A chain	F13A1	0.44	0.001412
P68103	Elongation factor 1-alpha 1	EEF1A1	9.07	0.015542					
Q2KJD0	Tubulin beta-5 chain	TUBB5	7.96	0.0180104					
Q862Q3	Beta-2-microglobulin	B2M	6.99	0.0198202					
Q9TTK6	Membrane primary amine oxidase	AOC3	6.28	0.0017246					
G5E5W1	Coagulation factor VIII	F8	5.93	0.0288047					
Q2KIW1	Paraoxonase 1	PON1	4.88	0.0258445					
F1MTT3	Coagulation factor XII	F12	4.57	0.0460769					
P62261	14-3-3 protein epsilon	YWHAE	4.30	0.0014777					
A0A3Q1MHU8	Complement factor properdin	CFP	3.63	0.0213611					
F1N102	Complement C8 beta chain	C8B	3.511	0.0392651					

Note: FC * stands for fold change (HS vs. Ctr).

## Data Availability

All data presented in this study are available upon reasonable request from the corresponding author.
